# Clonidine Use for the Treatment of Nightmares in Posttraumatic Stress Disorder

**DOI:** 10.1155/2022/5251406

**Published:** 2022-01-17

**Authors:** Jill S. Bange, Kelly E. Melvin

**Affiliations:** Department of Psychiatry and Behavioral Health, Marshall University, 1115 20th St. #205, Huntington, WV 25703, USA

## Abstract

**Introduction:**

While clonidine is used clinically for the treatment of nightmares in posttraumatic stress disorder (PTSD), few case reports demonstrating this indication exist, and there have been few studies investigating clonidine's mechanism of action for controlling nightmare symptoms. *Case Report.* In order to further characterize clonidine's role in treating nightmare symptoms in PTSD, we offer this case report describing one United States veteran who presented to an inpatient psychiatric unit after a suicide attempt. At that time, she described a remote history of PTSD symptoms, including nightmares, flashbacks, hyperarousal, and avoidance behaviors which had been well controlled on sertraline and clonidine. Upon her admission, her home sertraline and alprazolam were continued but her home clonidine was not continued. On day two of her hospital stay, she stated that her nightmares had returned. Her home clonidine was restarted on day two. On day three and thereafter, the patient no longer complained of nightmares.

**Conclusion:**

Our patient's nightmare symptoms had been controlled for years after beginning clonidine as an outpatient, but off clonidine, she had a return of her nightmare symptoms. Her nightmares again resolved once clonidine was resumed. Given this pattern in the patient's response to clonidine, this case may serve as additional evidence in the literature that clonidine has a role in treating nightmares in PTSD. Current proposed mechanisms of action for clonidine's ability to control nightmare symptoms in PTSD include that clonidine may alter the proportions of REM and non-REM sleep in a dose-dependent manner or that clonidine may play a role in memory consolidation. Further formal medication trials are the ideal future direction for establishing this role for clonidine.

## 1. Introduction

Posttraumatic stress disorder (PTSD) is an illness characterized by at least one month of intrusive recollections of trauma, such as in the form of nightmares or flashbacks, as well as avoidance behaviors, hyperarousal symptoms, and/or negative alterations in cognition or mood. These symptoms are present after experiencing or witnessing actual or threatened death, serious injury, or sexual violence, or learning that this event happened to a close relative or friend of the individual [1]. Dysregulation of the autonomic nervous system and sympathetic arousal secondary to alterations in norepinephrine basal level and exaggerated response to norepinephrine activation has been shown in the literature to play a role in the pathophysiology of PTSD [2–4].

Clonidine is an alpha-two agonist which acts on presynaptic receptors in the locus coeruleus [5, 6]. The alpha-two receptor is an autoreceptor, and agonism of this receptor decreases presynaptic calcium channel activity and therefore decreases norepinephrine release [7, 8]. Clonidine has uses in psychiatry for several conditions including opioid withdrawal, attention-deficit hyperactivity disorder (ADHD), Tourette syndrome, neuroleptic-induced akathisia, stimulant-induced insomnia, and clozapine-induced sialorrhea [9, 10]. Clonidine has also been studied with regard to improvement of several PTSD symptoms [11]. However, there has been little published in the literature regarding clonidine's efficacy in controlling nightmare symptoms in PTSD or proposed mechanisms for clonidine's ability to alleviate nightmare symptoms. One explanation in the literature is that clonidine may alter the amounts of rapid eye movement (REM) and non-REM sleep, increasing REM sleep stages at low doses and decreasing it at high doses [12, 13]. Another suggestion is that clonidine may play a role in the memory reconsolidation process [14]. Clonidine has been studied at doses of 0.1 mg to 4.0 mg for nightmares [1]. In order to further characterize clonidine's role in treating nightmare symptoms in PTSD, we offer this case report describing one United States veteran.

## 2. Patient Information

In May of 2021, a 42 year-old female with a history of bipolar 1 disorder presented to an inpatient psychiatric unit of a general medical hospital after attempting suicide using a home alprazolam prescription. She had been feeling hopeless after worsening relationship problems as well as after her youngest child had moved away to attend college. For the present episode, the patient reported depressed mood beginning one month prior associated with decreased sleep, low energy, diminished appetite, poor concentration, and suicidal ideation. In addition to recurrent episodes of major depression, our patient reported a history of discrete periods of elevated mood lasting one to two weeks which were associated with increased energy, risk-taking behavior, decreased need for sleep, increase in goal-directed activities, and pressured speech occurring one to two times per year. Her diagnosis of bipolar disorder was rendered utilizing the Diagnostic and Statistical Manual of Mental Disorders (DSM) 5. She had not had an episode of mania since her outpatient provider had started her on a mood-stabilizing agent several months prior. Due to the absence of mania symptomatology within the past 48 hours, a formal Young Mania Rating Scale was not completed as the score would have been zero. She had also described experiencing symptoms of PTSD in the past, including nightmares, flashbacks, hyperarousal, and avoidance behaviors which had not been bothersome to her for several years since sertraline and clonidine were added to her regimen, which had previously only included lamotrigine. In the past, the patient had experienced childhood abuse by her relatives. Further, she is an active duty US Army combat vet. During her time in military service, the patient stated that she witnessed many close friends die. Other relevant chronic medical conditions in this patient included asthma, type 2 diabetes mellitus, hypertension, and hypothyroidism. In addition to lamotrigine, sertraline, alprazolam, and clonidine, the patient was taking levothyroxine, hydrochlorothiazide, pantoprazole, and spironolactone at home; her diabetes was diet-controlled.

## 3. Clinical Findings and Diagnostic Assessment

When this patient first presented for admission, her mental status exam was significant for a disheveled appearance, restless psychomotor behavior, and dysphoric affect. Her speech, orientation, thought process, concentration, perception, and cognition were intact. Her thought content was notable for passive suicidal ideation and she exhibited fair insight and judgment. After initial evaluation, she was diagnosed with bipolar 1 disorder, current episode depressive, as well as posttraumatic stress disorder.

## 4. Therapeutic Intervention

Upon her admission, her home sertraline, lamotrigine, and alprazolam were continued but her home clonidine was not continued, as the patient could not remember the dose of her clonidine and the admitting physician was unable to verify the dose after hours. On day 2 of her hospital stay, she stated that the nightmares which contained events from her past trauma had returned, and she noted increased sweating upon waking in the morning. The dose of her clonidine was verified to be 0.15 mg at bedtime. Her home clonidine was restarted on day 2. On day 3 and thereafter, the patient no longer complained of nightmares.

## 5. Follow-Up and Outcomes

The patient chose to follow up with a psychiatric group which is not affiliated with the admitting hospital. During a phone call for the purposes of obtaining informed consent for this publication five months post discharge, the patient stated that she continued to have absence of her nightmares while taking her clonidine, and that overall she was doing well.

## 6. Discussion

Although clonidine has been studied for multiple psychiatric disorders, few studies have examined its use for nightmare symptoms specifically. One open-label trial reported improvement for subjective sleep complaints on clonidine, but not nightmares specifically [15]. A case report of an 11 year-old pediatric patient with PTSD described improvement of nightmare symptoms [16]. Another case report described two war veterans with PTSD, one with a traumatic brain injury, who both had resolution of nightmare symptoms once clonidine was initiated [17]. Our patient's nightmare symptoms had been controlled for years after beginning clonidine as an outpatient, but off clonidine, she had a return of her nightmare symptoms. Her nightmares again resolved once clonidine was resumed. Given this pattern in the patient's response to clonidine, this case may serve as additional evidence in the literature that clonidine has a role in treating nightmares in PTSD.

A review of nightmare treatment in PTSD patients at one Veterans Affairs hospital discovered that out of 27 articles, 63% described clonidine to partially relieve nightmare symptoms and 37% described clonidine to have no effect on nightmare symptoms [1]. Compared to prazosin, another agent which influences the alpha adrenergic system, in this case by blocking alpha 1 receptors, clonidine is much less studied with respect to its effect on nightmare symptoms [1].

A clear mechanism of action for clonidine's ability to control nightmare symptoms does not appear to have been identified in the literature to date. Others have speculated this may be because clonidine seems to alter the proportions of REM and non-REM sleep in a dose-dependent manner. In a study by Miyazaki et al., low-dose clonidine (0.025 mg) significantly decreased non-REM sleep duration and increased REM sleep duration. Medium-dose clonidine (0.15 mg) increased non-REM sleep duration and decreased REM sleep duration, and additionally elongated REM latency. REM sleep generation is believed to be permitted by the decreased activity of noradrenergic neurons in the locus coeruleus. It was proposed that at the lower dose, clonidine preferentially bound presynaptic alpha-2 receptors on noradrenergic neurons in the locus coeruleus, permitting REM sleep. However, at the higher dose, clonidine is believed to also bind postsynaptic alpha-2 receptors, acting as an autoreceptor and reducing norepinephrine release at the locus coeruleus, thus decreasing REM sleep [12, 13]. Another proposed mechanism is that clonidine may play a role in memory consolidation. In rats, clonidine was found to decrease the freeze response to an auditory tone which had been conditioned to be associated with a shock stimulus. It was suggested that clonidine disrupted the reconsolidation process of fear memories, impairing the rats' ability to associate the auditory tone with the shock stimulus [14].

These possible mechanisms may offer partial explanations for clonidine's role in decreasing nightmares. However, a change in REM sleep duration might indicate that incidence of dreaming overall, rather than just nightmares, would be impacted. Clonidine is not known to decrease the amount of dreaming overall or have preferential efficacy for trauma-themed nightmares over nightmares with other content. Additionally, whether clonidine has an impact on memory consolidation would not necessarily explain its role in controlling nightmares specifically. Further formal medication trials are the ideal future direction for establishing this role for clonidine.
